# Targeting immune co-stimulatory effects of PD-L1 and PD-L2 might represent an effective therapeutic strategy in stroke

**DOI:** 10.3389/fncel.2014.00228

**Published:** 2014-08-11

**Authors:** Sheetal Bodhankar, Yingxin Chen, Andrew Lapato, Arthur A. Vandenbark, Stephanie J. Murphy, Halina Offner

**Affiliations:** ^1^Neuroimmunology Research, Portland Veterans Affairs Medical CenterPortland, OR, USA; ^2^Department of Neurology, Oregon Health and Science UniversityPortland, OR, USA; ^3^Department of Anesthesiology and Perioperative Medicine, Oregon Health and Science UniversityPortland, OR, USA; ^4^Department of Molecular Microbiology and Immunology, Oregon Health and Science UniversityPortland, OR, USA

**Keywords:** MCAO, co-stimulatory pathway, programmed death ligand-1 and 2, T-cells, regulatory B cells

## Abstract

Stroke outcome is worsened by the infiltration of inflammatory immune cells into ischemic brains. Our recent study demonstrated that PD-L1- and to a lesser extent PD-L2-deficient mice had smaller brain infarcts and fewer brain-infiltrating cells vs. wild-type (WT) mice, suggesting a pathogenic role for PD-ligands in experimental stroke. We sought to ascertain PD-L1 and PD-L2-expressing cell types that affect T-cell activation, post-stroke in the context of other known co-stimulatory molecules. Thus, cells from male WT and PD-L-deficient mice undergoing 60 min of middle cerebral artery occlusion (MCAO) followed by 96 h of reperfusion were treated with neutralizing antibodies to study co-stimulatory and co-inhibitory interactions between CD80, cytotoxic T-lymphocyte antigen-4 (CTLA-4), PD-1, and PD-Ls that regulate CD8^+^ and CD4^+^ T-cell activation. We found that antibody neutralization of PD-1 and CTLA-4 signaling post-MCAO resulted in higher proliferation in WT CD8^+^ and CD4^+^ T-cells, confirming an inhibitory role of PD-1 and CTLA-4 on T-cell activation. Also, CD80/CD28 interactions played a prominent regulatory role for the CD8^+^ T-cells and the PD-1/PD-L2 interactions were dominant in controlling the CD4^+^ T-cell responses in WT mice after stroke. A suppressive phenotype in PD-L1-deficient mice was attributed to CD80/CTLA-4 and PD-1/PD-L2 interactions. PD-L2 was crucial in modulating CD4^+^ T-cell responses, whereas PD-L1 regulated both CD8^+^ and CD4^+^ T-cells. To establish the contribution of PD-L1 and PD-L2 on regulatory B-cells (Bregs), infarct volumes were evaluated in male PD-L1- and PD-L2-deficient mice receiving IL-10^+^ B-cells 4h post-MCAO. PD-L2- but not PD-L1-deficient recipients of IL-10^+^ B-cells had markedly reduced infarct volumes, indicating a regulatory role of PD-L2 on Bregs. These results imply that PD-L1 and PD-L2 differentially control induction of T- and Breg-cell responses after MCAO, thus suggesting that selective targeting of PD-L1 and PD-L2 might represent a valuable therapeutic strategy in stroke.

## INTRODUCTION

Stroke is a leading cause of death and disability worldwide. The most common type of stroke is ischemic stroke (87% of cases), where an infarct develops after a few minutes of ischemia (Lakhan et al., 2009). Ischemic stroke is characterized by the disruption of cerebral blood flow (Dirnagl et al., 1999) and a timely restoration of blood flow (reperfusion) achieved by intravenous administration of tissue plasminogen activator within 4.5 h after stroke onset remains the only approved treatment for limiting brain injury following ischemic stroke. While on the one hand, reperfusion of the ischemic brain is desirable, on the other it may lead to tissue damage. Reperfusion is known to enhance the inflammatory response and causes additional injury to adjacent brain tissue (Schaller and Graf, 2004). Several factors have been described as mediators of ischemia–reperfusion (I–R)-induced brain injury (Iadecola and Anrather, 2011; Macrez et al., 2011). Although the cells of the innate immune system, especially neutrophils and monocytes, always seem to be in focus, recent studies have demonstrated that T-cells also have an impact on tissue damage.

In fact, several studies evaluating stroke outcome in T-cell-deficient mice have consistently reported a smaller infarct volume and improved functional outcome than in wild-type (WT) controls (Yilmaz et al., 2006; Hurn et al., 2007; Shichita et al., 2009; Kleinschnitz et al., 2010). Studies also demonstrate that mice lacking RANTES (CCL5), a chemokine that recruits T-cells (as well as other immune cells) into inflammatory sites (Appay and Rowland-Jones, 2001), have smaller infarct volumes than WT mice, again attributing a pathogenic role to the T-cells. However, the mechanisms of T-cell-mediated brain injury following stroke remain unclear. It is also not known whether the activation of T-cells follows the classical antigen-dependent pathway or if the infiltration into the brain following stroke is too rapid to follow the classical pathway.

The classical antigen-dependent activation of naive T-cells comprises two main steps (Abbas, 2011). The first step involves the binding of the T-cell receptor (TCR) to the antigen presented on the major histocompatability complex on the surface of an antigen-presenting cell (APC). The second step involves the binding of co-stimulatory molecules on the T-cell and the APC, such as CD28 on the T-cell and CD80 (B7.1) and CD86 (B7.2) on the APC (Santana and Rosenstein, 2003). This second step involving co-receptor signaling is an important mechanism for coordinating and tightly regulating immune responses. Co-stimulation via ligation of the co-receptor CD28 on T-cells by B7 molecules on APCs is required for optimal T-cell activation (Linsley et al., 1991). Once mobilized, however, T-cells begin to express other members of the CD28/B7 receptor families that attenuate the immune response through inhibition of proliferation and cytokine production (Peggs et al., 2009). Among the negative signaling molecules, cytotoxic T-lymphocyte antigen-4 (CTLA-4) and PD-1 belonging to the CD28/B7 families, are the most studied. CTLA-4 is rapidly up-regulated following T-cell activation and binds to B7 molecules (Curran et al., 2010). PD-1 (CD279 in CD nomenclature) is expressed on activated T and B cells as well as on activated myeloid cells and both these co-receptors elicit inhibitory signals upon co-ligation with the TCR and play a role in the control of tolerance (Ishida et al., 1992; Agata et al., 1996; Greenwald et al., 2005). Two PD-1 ligands have thus far been described, PD-L1 also named as B7-H1 (CD274), and PD-L2 also named as B7-DC (CD273; Dong et al., 1999; Latchman et al., 2001). The expression of PD-L1 within non-lymphoid tissues suggests that it may regulate the function of self-reactive immune cells in peripheral tissues and thus, may regulate inflammatory responses (Keir et al., 2006). In addition to PD-1, studies have demonstrated that PD-L1 also interacts with CD80 in both mice and humans (Butte et al., 2007, 2008). Both PD-L1 and PD-L2 inhibit T-cell proliferation, cytokine production and cell adhesion (Latchman et al., 2001; Saunders et al., 2005), although some contradictory data have suggested a co-stimulatory function (Dong et al., 1999).

Prior research from our laboratory, performed using PD-1^-/-^ mice, demonstrated the inhibitory effects of PD-1 in stroke because when MCAO was compared to WT male mice after 96 h of reperfusion, cortical, striatal, and total infarct volumes were significantly larger in PD-1^-/-^ mice, with a marked recruitment of inflammatory cells from the periphery into the central nervous system (CNS; Ren et al., 2011). Studies were then extended to investigate the role of the PD-ligands, PD-L1 and PD-L2, in modulating severity of ischemic brain injury and the associated CNS inflammation. Contrary to our expectations, PD-L1-deficient (PD-L1^-/-^) and PD-L2-deficient (PD-L2^-/-^) mice that were similarly subjected to 60 min of MCAO followed by 96 h of reperfusion demonstrated smaller total infarct volumes compared to WT mice (Bodhankar et al., 2013). The immune parameters matched the stroke outcome in that the PD-L1^-/-^, and to a lesser extent PD-L2^-/-^ mice, had reduced levels of proinflammatory activated microglia and/or infiltrating monocytes and CD4^+^ T-cells in the ischemic hemispheres, thus suggesting a pathogenic rather than a regulatory role for both PD-ligands. Knowing that T-cell influx is highly time-dependent, a more thorough characterization of the temporal profile of various T-cell subsets was needed. Therefore, discerning the mechanisms that lead to T-cell activation in the periphery and subsequent proliferation and mobilization of T-cells into the CNS is a very critical determinant in ischemic stroke outcome.

Hence, the aim of the current study was to assess the contribution of the various co-stimulatory molecules in controlling T-cell proliferation as well as to verify the PD-L-expressing cell-types responsible for mediating its central effects after MCAO. For this purpose, T-cells obtained from WT, PD-L1^-/-^ and PD-L2^-/-^ mice were carboxyfluorescein succinimidyl ester (CFSE)-labeled and co-cultured with APCs in the presence of various neutralizing antibodies (Abs) to co-stimulatory receptors. Our results demonstrate that antibody neutralization of PD-1 and CTLA-4 signaling after MCAO resulted in higher proliferation levels in WT CD8^+^ and to a lesser extent CD4^+^ T-cells, confirming an inhibitory role of PD-1 and CTLA-4 on T-cell activation. Conversely, antibody neutralizing of CD80 and PD-L1 after MCAO resulted in reduced proliferation, indicating a stimulatory role of CD80 and PD-L1 in WT mice on T-cell activation. Interestingly, the CD80/CD28 interactions appeared to play a prominent regulatory role for the CD8^+^ T-cells while the PD-1/PD-L2 interactions were dominant in controlling the CD4^+^ T-cell responses in WT mice, after stroke. The suppressive phenotype in PD-L1 deficient mice was attributed to CD80/CTLA-4 and PD1/PD-L2 interactions. PD-L2 was crucial in modulating CD4^+^ T-cell responses, whereas PD-L1 predominantly regulated CD8^+^ T-cells. To establish the contribution of PD-L1 and PD-L2 on regulatory B-cells (Bregs), infarct volumes were evaluated in male PD-L1- and PD-L2-deficient mice receiving IL-10^+^ B-cells 4 h after MCAO. PD-L2- but not PD-L1-deficient recipients of IL-10^+^ B-cells had markedly reduced infarct volumes, indicating a regulatory role of PD-L2 on Bregs. These results imply that PD-L1 and PD-L2 differentially control induction of T- and Breg-cell responses after MCAO.

## MATERIALS AND METHODS

### ANIMALS

PD-L1-deficient (PD-L1^-/-^) and PD-L2-deficient (PD-L2^-/-^) mice on the C57BL/6 background were gifts from Indira Guleria, PhD (Transplantation Research Center, Brigham and Women’s Hospital, Children’s Hospital Boston, and Harvard Medical School, Boston, MA, USA) and Arlene Sharpe, Ph.D (Department of Pathology, Harvard Medical School, Boston, MA, USA), respectively. Based on Dr. Guleria’s and Dr. Sharpe’s recommendations and previous publications (Keir et al., 2006; Zhang et al., 2010), age-matched 8–12-week-old male were used. WT C57BL/6J mice were obtained from Jackson Laboratory, Bar Harbor, ME, USA. Animals were randomized to treatment groups. All experiments were performed in accordance with National Institutes of Health (NIH) guidelines for the use of experimental animals, and the protocols were approved by Portland Veteran Affairs Medical Center and Oregon Health & Science University Animal Care and Use Committees.

### MIDDLE CEREBRAL ARTERY OCCLUSION (MCAO) MODEL

All surgeries were conducted under aseptic conditions by a single surgeon. Transient focal cerebral ischemia was induced in male PD-L1^-/-^, PD-L2^-/-^, and WT mice for 60 min by reversible right MCAO under isoflurane anesthesia followed by 96 h of reperfusion as previously described (Chen et al., 2012). Head and body temperatures were controlled at 36.5 ± 1.0°C before, during, and after MCAO with warm water pads and a heating lamp. The common carotid artery was temporarily occluded and a 6-0 nylon monofilament surgical suture (Ethicon, Somerville, NJ, USA) with a silicone-coated (Xantopren Comfort Light, Heraeus, Hanau, Germany) tip was inserted via an external carotid artery stump distal to the internal carotid artery to the origin of the middle cerebral artery. After 60 min of MCAO, reperfusion was initiated by intraluminal filament withdrawal and the incision was closed with 6-0 surgical sutures (Ethicon). Each animal was then awakened and recovered in a separate cage with a warm water pad. In sham-treated mice, the filament was placed but not advanced to achieve MCAO. Occlusion and reperfusion were verified in each animal by laser Doppler flowmetry (LDF; Model DRT4, Moor Instruments, Wilmington, DE, USA). Animals were excluded if intra-ischemic LDF (percentage of pre-ischemic LDF baseline) was greater than 30%. Neurological deficit scores were determined at 1, 24, 48, 72, and 96 h of reperfusion to confirm ischemia and the presence of ischemic injury using a 0 to 4-point scale as follows: 0, no neurological dysfunction; 1, failure to extend left forelimb fully when lifted by tail; 2, circling to the contralateral side; 3, falling to the left; and 4, no spontaneous movement or in a comatose state (Chen et al., 2012). The surgeon was not blinded to the animal genotypes. However, the surgeon was blinded to treatment groups.

### CELL ISOLATION

Splenocyte suspensions were prepared in Roswell Park Memorial Institute (RPMI) 1640 by mechanical disruption followed by use of red cell lysis buffer (eBioscience, San Diego, CA, USA) according to the manufacturer’s instructions. The cells were washed twice with RPMI-1640, counted, and resuspended in stimulation medium containing 2% fetal bovine serum (FBS; HyClone, GE Healthcare, UT, USA).

### PURIFICATION AND ISOLATION OF T-CELLS

T-cells from splenocytes of MCAO-subjected WT, PD-L1^-/-^ and PD-L2^-/-^ mice were purified using paramagnetic bead-conjugated Abs from the pan T-cell isolation kit II and subsequently separated by AutoMACS (Miltenyi Biotec, Auburn, CA, USA), according to manufacturer’s instructions. The purity of the negative fraction of cells thus separated (CD3^+^ T-cells) was examined by flow cytometry. Cell preparations demonstrating >98% purity of each cell type were used for co-culture studies.

### CFSE LABELING AND T-CELL PROLIFERATION ASSAY

Sorter-purified T-cells were washed twice in PBS, labeled with 2.5 μM CFSE (Molecular Probes, Eugene, OR, USA), for 7 min at 37°C and quenched with stimulation medium containing 10% FBS. Cells were washed twice in stimulation medium containing 2% FBS before they were plated for the proliferation assay. CFSE-labeled purified T-cells (1 × 10^5^/well) were co-cultured with unlabeled non-T-cells (1 × 10^5^/well) in 96-well plates and were incubated in the presence of anti-CD3 Ab [2.5 μg/mL; baseline condition (purified hamster anti-mouse CD3ε; clone 145-2C11)] for 72 h. Functional grade purified neutralizing Abs to CD80 (clone 16-10A1), CD152 (CTLA-4, clone 9H10), CD279 (PD-1; clone RMP1-14), CD274 (PD-L1; clone MIH5), CD273 (PD-L2, clone TY25), and CD28 (clone 37.51) were used at a final concentration of 10 μg/mL, alone or in combination with other neutralizing Abs (eBioscience, San Diego, CA, USA). Proper negative control conditions such as T-cells + non T-cells with no anti-CD3 Ab and non-CFSE-labeled T-cells were also included. Each culture condition was plated in triplicate (or in some cases where very few splenocytes were acquired after stroke, in duplicate). After 3 days, CD8^+^ and CD4^+^ T-cell proliferation was assessed based on CFSE dilution and analyzed by flow cytometry. Each experiment was repeated at least twice.

Inhibition of T-cell proliferation in the presence of non-T-cells was calculated from the following formula:

Stimulation index (SI) = [percent proliferating cells in presence of neutralizing Ab] – [percent expression at baseline]/[percent expression at baseline].

### CELL SORTING AND ADOPTIVE TRANSFER OF B-CELLS

Male IL-10 GFP reporter mice served as donors of B-cells. Splenic CD19^+^ B-cells were purified using paramagnetic bead-conjugated Abs from the CD19 cell isolation kit and subsequently separated by AutoMACS^TM^ (Miltenyi Biotec, Auburn, CA, USA). The negative fraction of the cells thus separated were CD19^+^ B-cells with a purity of ≥94%. CD19^+^ B-cells were suspended in RPMI 1640 medium with 2% FBS and cultured in the presence of 1 μg/mL lipopolysaccharide (LPS, *Escherichia coli* strain K12) for 48 h. After 48 h of culture, B-cells were harvested from culture plates, washed free of LPS and viable cells were counted using a hemocytometer with the trypan blue exclusion method. Five million purified IL-10-GFP^+^ B-cells from the donor mice were suspended in 100 μL RPMI 1640 medium and were transferred intravenously (i.v.) into PDL1^-/-^ and PD-L2^-/-^ mouse experimental groups 4 h after MCAO. Each PDL1^-/-^ and PD-L2^-/-^ mouse received either 5 × 10^6^/100 μL purified IL-10-GFP^+^ B-cells or 100 μL RPMI 1640 medium (control group).

### INFARCT VOLUME ANALYSIS

The individual performing infarct volume analysis was not blinded to genotype but was blinded to the treatment groups. Mice were euthanized and brains collected at 96 h of reperfusion for 2,3,5-triphenyltetrazolium chloride histology and then digital image analysis of infarct volume was undertaken as previously published (Chen et al., 2012). Images were analyzed using SigmaScan Pro 5.0 (Systat Software, Inc., Point Richmond, CA, USA). To control for edema, regional infarct volume (cortex, striatum, and hemisphere) was determined by subtraction of the ipsilateral non-infarcted regional volume from the contralateral regional volume. This value was then divided by the contralateral regional volume and multiplied by 100 to yield regional infarct volume as a percentage of the contralateral region.

### ANALYSIS OF CELL POPULATIONS BY FACS

The individual performing FACS analysis was not blinded to genotype. Anti-mouse Abs CD4 (GK1.5, BD Pharmingen, Franklin Lakes, NJ, USA) and CD8 (53-6.7, BD Pharmingen) were used for the proliferation assay. Anti-mouse CD19 (1D3, BD Pharmingen), CD1d (1B1, BD Pharmingen), CD5 (53-7.3, BD Pharmingen), CD28 (37.51, BD Pharmingen), CD152 (CTLA-4, UC10-4B9), ICOS (C398-4A, BD Pharmingen), PD-L1 (MIH5, eBioscience), and PD-L2 (TY25, eBioscience) were used for this study. Single-cell suspensions were washed with staining medium (PBS containing 0.1% NaN_3_ and 2% FCS). After incubation with mAb and washing with staining buffer, propidium iodide (PI) was added to identify dead cells. FACS data acquisition was performed on a FACSCalibur flow cytometer (BD Biosciences, San Jose, CA, USA) and data were analyzed using isotype control Abs to set quadrants before calculating the percentage of positive cells, using FCS Express (De Novo Software, Los Angeles, CA, USA).

### INTRACELLULAR STAINING

Intracellular staining was visualized using a published immunofluorescence protocol (Subramanian et al., 2011). Briefly, 2 × 10^6^ cells/mL were resuspended in complete medium (RPMI-1640 containing 10% FCS, 1 mM/L pyruvate, 200 μg/mL penicillin, 200 U/mL streptomycin, 4 mM/L L-glutamine, and 5 × 10^-5^ mol/L 2-β-ME), with PMA (50 ng/mL), ionomycin (500 ng/mL), and Brefeldin A (10 μg/mL, Sigma–Aldrich) for 4 h. For intracellular IL-10 detection, a modification was followed for the immunofluorescence staining protocol (Yanaba et al., 2008). Briefly, isolated leukocytes or purified cells were resuspended (2 × 10^6^ cells/mL) in complete medium and cultured with LPS (10 μg/mL) in addition to PMA (50 ng/mL), ionomycin (500 ng/mL), and Brefeldin A (10 μg/mL; all reagents from Sigma-Aldrich) for 4 h. Fc receptors were blocked with anti-FcR mAb (2.3G2, BD Pharmingen) before cell surface staining, fixed, and permeabilized with the Fixation/Permeabilization buffer (eBioscience), according to the manufacturer’s instructions. Permeabilized cells were washed with 1× Permeabilization Buffer (eBioscience) and stained with APC-conjugated anti-IL-10 mAb (JES5-16E3, eBioscience). Isotype matched mAb served as negative controls to demonstrate specificity and to establish background IL-10 staining levels.

### STATISTICAL ANALYSIS

All values are reported as mean ± SEM. For flow data analysis and representation of three and more groups, the one-way ANOVA followed by *post hoc* Tukey’s test was applied. For the proliferation assay, the one-way ANOVA with Dunnett’s *post hoc* test to compare all neutralizing Ab conditions to just the anti-CD3 Ab treated (Control) value was applied. Statistical analyses were performed using GraphPad PRISM software version 5 (La Jolla, CA, USA). For all tests, *p* values ≤0.05 were considered statistically significant. Significant differences are denoted as ^∗^*p* ≤ 0.05; ^∗∗^*p* ≤ 0.01; ^∗∗∗^*p* ≤ 0.001. Differences in cortical, striatal, and hemispheric (total) infarct volumes were determined by one-way ANOVA with *post hoc* Newman–Keul’s test. Statistical significance was *p* < 0.05. Statistical analyses for infarct volumes were performed using SigmaStat Statistical Software, Version 3.1 (SPSS Inc., Chicago, IL, USA).

## RESULTS

### CD80/CD28 INTERACTIONS PROMINENTLY REGULATE CD8^+^ T-CELLS, WHILE PD-1/PD-L2 CONTROL THE CD4^+^ T-CELL RESPONSES IN WT MICE SUBJECTED TO MCAO

Over the past few years, our laboratory has demonstrated an inhibitory role for PD-1 in ischemic stroke (Ren et al., 2011), but on the contrary, a co-stimulatory role for PD-L1 and to a lesser extent PD-L2 (Bodhankar et al., 2013). To clarify these findings, it was important to discern which combinations of the major co-stimulatory molecules and their known binding partners might contribute to stroke outcome.

**FIGURE 1:**
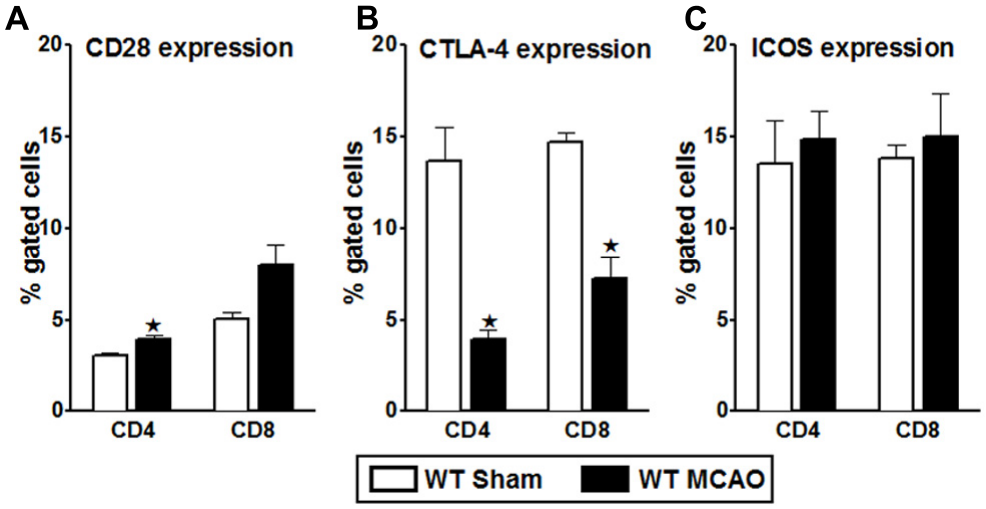
**Characterization of co-stimulatory molecules on T-cells of WT splenocytes, post-MCAO.** Splenocytes from sham- and MCAO-subjected WT mice were harvested 96 h after MCAO (60 min) and assessed for expression of: **(A)** CD28; **(B)** CTLA-4; and **(C)** ICOS on gated CD4^+^ and CD8^+^ T-cells. Values represent mean numbers (±SEM) of indicated cell subsets, gated on live leukocytes (by PI exclusion), from 4–5 mice of each group, from two separate experiments. Statistical analysis was performed using Student’s *t-*test. Significant differences between sample means are indicated as *p ≤ 0.05 as compared to the sham-treated WT mice.

Though the PD-1/PD-L pathway is important in governing T-cell activation and proliferation, it is not the only determinant pathway. Hence, to assess the role played by various other co-stimulatory pathways potentially involved in T-cell activation/proliferation after MCAO, neutralizing Abs were used. However, to narrow down the combinations of neutralizing Abs needed, we first identified key players in the co-stimulatory pathway on splenocytes from WT mice subjected to 60 min of MCAO followed by 96 h of reperfusion in comparison with sham mice. As demonstrated in **Figure 1A**, the expression of the positive co-stimulatory molecule, CD28, was significantly increased on CD4^+^ T-cells after MCAO, with a similar trend for its increased expression on CD8^+^ T-cells. On the other hand, the expression of the negative co-stimulatory molecule, CTLA-4, which opposes the actions of CD28-mediated co-stimulation (Greenwald et al., 2005), was significantly down regulated both on CD4^+^ and CD8^+^ splenic T-cells (**Figure 1B**). We also determined the expression levels of ICOS, which is known to synergize with CD28 to promote the activation of T-cell responses (Greenwald et al., 2005). However, no difference in the level of expression of ICOS as compared to the sham MCAO-subjected WT splenocytes, was demonstrated (**Figure 1C**).

**FIGURE 2:**
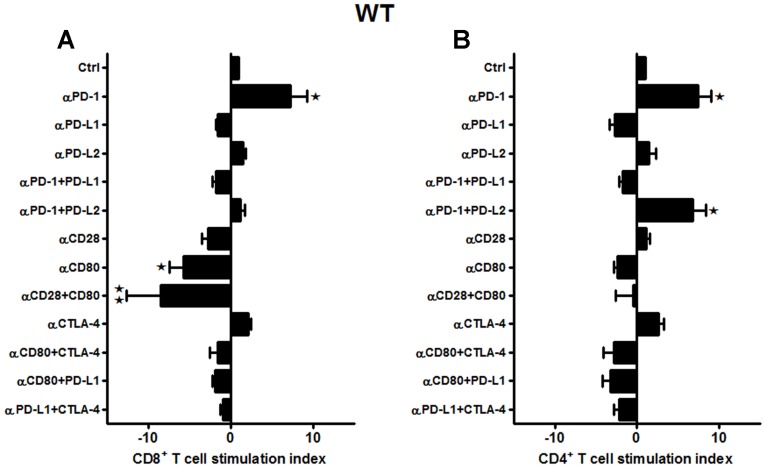
**CD80/CD28 interactions prominently regulate CD8^+^ T-cells, while PD-1/PD-L2 control the CD4^+^ T-cell responses in WT mice subjected to MCAO.** T-cells were purified, by negative sort, from the spleens of MCAO-subjected WT mice, by labeling with specific microbeads and separating on the AutoMACS^TM^. The purified T-cells were CSFE-labeled and then cultured with non-T-cells from the same mice at a 1:1 ratio (T:APC) in the presence of anti-CD3 antibody (2.5 μg/mL; baseline) and other neutralizing Abs (10 μg/mL) to co-stimulatory molecules, in 96-well plates. After 72 h of culture, cells were washed and evaluated by FACS Calibur for **(A)** CD8^+^ and **(B)** CD4^+^ T-cell expression and CFSE dilution. Data represent the stimulation indices of the CD8 and CD4 T-cells in the presence of neutralizing Abs as compared to the control (T:APC + anti-CD3 Ab) condition). The data are represented such that the baseline value is 1 and all other values are adjusted relative to the baseline. Data presented are representative of splenocytes obtained from nine WT mice, with least three separate experiments and each experiment comprising duplicates or triplicates of the given neutralizing Ab condition. Significant differences between sample means are indicated as *p ≤ 0.05, **p ≤ 0.01 as compared to the baseline condition.

Furthermore, neutralizing Abs to the classic B7/CD28, B7/CTLA-4, and the PD-1/PD-L co-stimulatory receptors were evaluated in WT mice subjected to 60 min MCAO followed by 96 h of reperfusion. Splenocytes from MCAO-subjected WT mice were obtained and total T-cells, sorted by negative selection, were labeled with CFSE. These labeled T-cells were then co-cultured with APCs in the presence of different combinations of neutralizing Abs to various co-stimulatory molecules. After 72 h of co-culture, the CD8^+^ and CD4^+^ T-cell proliferative capacities were determined by CFSE dilution by flow cytometry. As demonstrated in **Figure 2A**, proliferation of CD8^+^ T-cells, post MCAO, was significantly reduced in the presence of anti-CD80 Ab (*p* ≤ 0.05), and more so when both anti-CD28 and anti-CD80 Abs were used in combination (*p* ≤ 0.01). There was also a trend towards decreased proliferation using anti-PD-L1 and anti-CD28 Abs, individually and in combination, although the changes were not significant. These changes indicate a positive role for the CD28/CD80 co-stimulatory molecules in promoting CD8^+^ T-cell proliferation in WT mice subjected to MCAO. On the other hand, neutralizing PD-1 led to a significant increase (*p* ≤ 0.05) in proliferation of CD8^+^ T-cells. Similarly, neutralizing only PD-L2 or CTLA-4 led to nominally but not significantly increased proliferation of CD8^+^ T-cells. Thus, only PD-1 could be clearly implicated as a negative regulator of CD8^+^ T-cell proliferation in MCAO-subjected WT mice.

Interestingly, a different pattern of proliferative capacities was demonstrated for CD4^+^ T-cells as compared to the CD8^+^ T-cells obtained from the WT mice in response to various neutralizing Abs (**Figure 2B**). The CD4^+^ T-cells also demonstrated nominally lower proliferative capacities in the presence of anti-CD80 and anti-PD-L1 neutralizing Abs, but there were no combinations tested that produced significantly reduced responses and thus the identification of a single set of co-stimulatory molecules. The CD4^+^ T-cells had significantly increased proliferation responses in the presence of anti-PD-1 alone or in combination with anti-PD-L2 but not anti-PD-L1 neutralizing Abs. Thus to summarize, CD28/CD80 interactions appear to play a prominent co-stimulatory role in proliferation of WT CD8^+^ but not CD4^+^ T-cells, whereas PD-1 functions as a negative regulator of both CD8^+^ and CD4^+^ T-cell proliferation in MCAO-treated mice. However, this regulation is permitted by co-expression of PD-L2 but not PD-L1, thus implicating a PD-1/PD-L2 co-inhibitory pathway for CD4^+^ T-cell proliferation in MCAO.

### NEUTRALIZING THE MAJOR CO-STIMULATORY MOLECULES IN THE PD-L1^-/-^ MICE, SUBJECTED TO MCAO, LEADS TO AN INCREASE IN BOTH CD8^+^ AND CD4^+^ T-CELL PROLIFERATIVE CAPACITIES, INDICATING AN OVERALL SUPPRESSIVE PHENOTYPE

**FIGURE 3:**
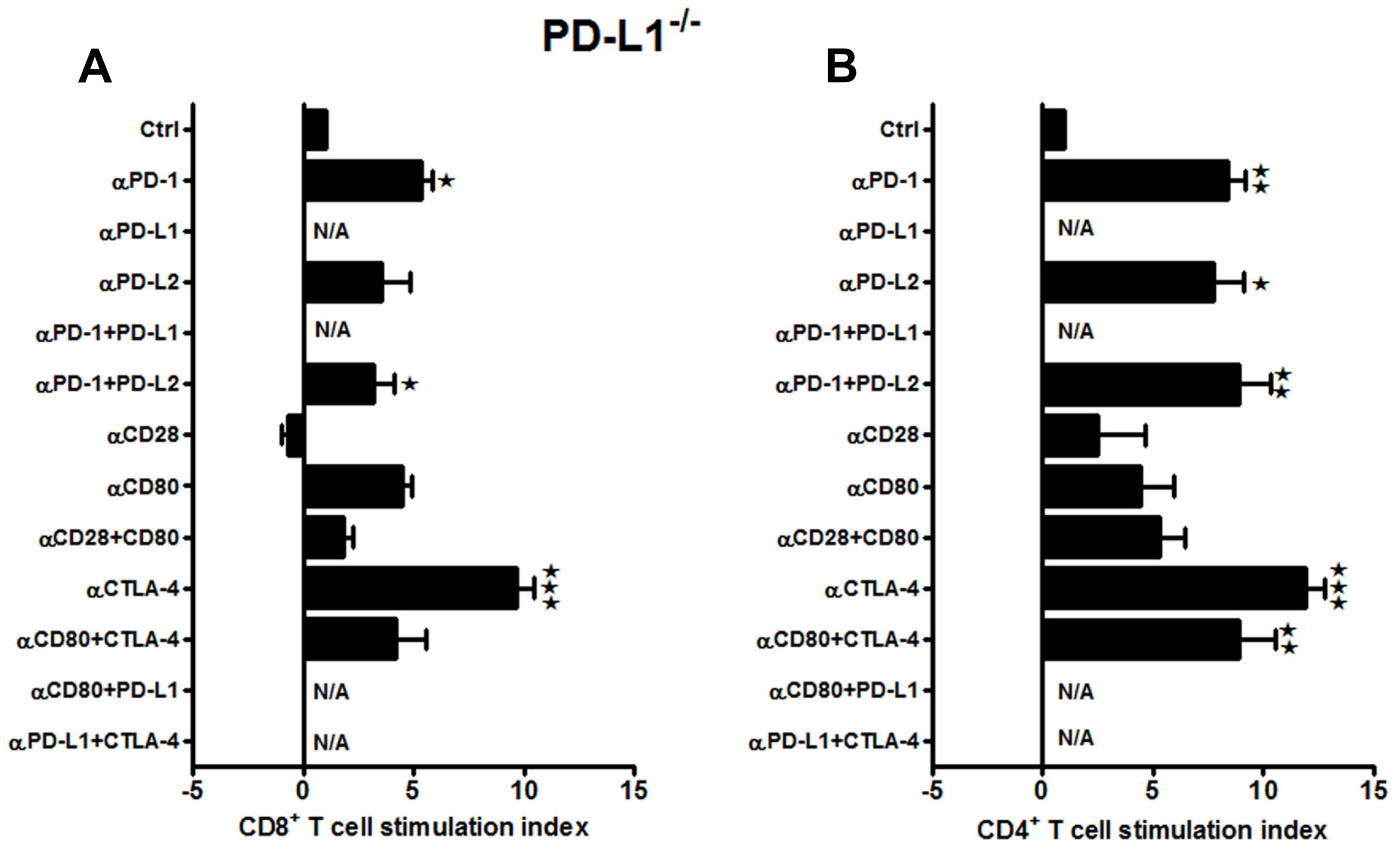
**Neutralizing the major co-stimulatory molecules in PD-L1^-/-^ mice, subjected to MCAO, leads to an increase in both CD8^+^ and CD4^+^ T-cell proliferative capacities, indicating an overall suppressive phenotype.** T-cells were purified, by negative sort, from the spleens of MCAO-subjected PD-L1^-/-^ mice, by labeling with specific microbeads and separating on the AutoMACSTM^TM^. The purified T-cells were CSFE-labeled and then cultured with non-T-cells from the same mice at a 1:1 ratio (T:APC) in the presence of anti-CD3 antibody (2.5 μg/mL; baseline) and other neutralizing Abs (10 μg/mL) to co-stimulatory molecules, in 96-well plates. After 72 h of culture, cells were washed and evaluated by FACS Calibur for **(A)** CD8^+^ and **(B)** CD4^+^ T-cell expression and CFSE dilution. Data represent the stimulation indices of the CD8 and CD4 T-cells in the presence of neutralizing Abs as compared to the control [(T:APC + anti-CD3 Ab) condition]. The data are represented such that the baseline value is 1 and all other values are adjusted relative to the baseline. Data presented are representative of splenocytes obtained from six PD-L1^-/-^ mice, with least three separate experiments and each experiment comprising duplicates or triplicates of the given neutralizing Ab condition. Significant differences between sample means are indicated as *p ≤ 0.05, ** *p* ≤ 0.01 and ***p ≤ 0.001 as compared to the baseline condition.

Our previous work demonstrated a more significant reduction in infarct volumes in PD-L1^-/-^ vs. PD-L2^-/-^ mice as compared to the WT mice (Bodhankar et al., 2013). Therefore, it was crucial to decipher the contributing co-stimulatory molecules leading to a suppressive phenotype in the absence of PD-L1. Total T-cells were purified from the spleens of PD-L1^-/-^ mice, labeled with CFSE and co-cultured with APCs in presence of neutralizing Abs to various co-stimulatory molecules for 72 h. In the absence of PD-L1 as in WT mice, the CD8^+^ T-cells exhibited significantly enhanced proliferation with anti-PD-1 (*p* ≤ 0.05) as well as the combination of anti-PD-1 + anti-PD-L2 Abs (*p* ≤ 0.05; **Figure 3A**). These results suggest involvement of PD-1/PD-L2 interactions and provide further support for the lack of regulatory PD-1/PD-L1 interactions. Moreover, anti-CTLA-4 (*p* ≤ 0.001) also promoted CD8^+^ T-cell proliferation, thus suggesting emergence of this co-inhibitory molecule in the absence of PD-L1. Importantly, neutralizing CD28 and CD80 alone or in combination did not result in loss of CD8^+^ T-cell proliferation as in WT mice, thus suggesting that PD-L1 expression enables CD28/CD80 co-stimulation. Use of neutralizing Abs also had a remarkably pronounced effect on the splenic CD4^+^ T-cells obtained from the MCAO-subjected PD-L1^-/-^ mice (**Figure 3B**). In the absence of PD-L1, neutralizing PD-1 and PD-L2 receptors, individually (*p* ≤ 0.01 and *p* ≤ 0.05, respectively) as well as in combination (*p* ≤ 0.01), demonstrated a significant increase in proliferative capacities. Also a significant increase in proliferation was observed by neutralizing just CTLA-4 (*p* ≤ 0.001) and the combination of CTLA-4 + CD80 (*p* ≤ 0.01). These results re-confirm a regulatory role for the PD-1/PD-L2 pathway and as well, establish a regulatory function for the CTLA-4/CD80 pathway that may emerge in the absence of PD-L1.

**FIGURE 4:**
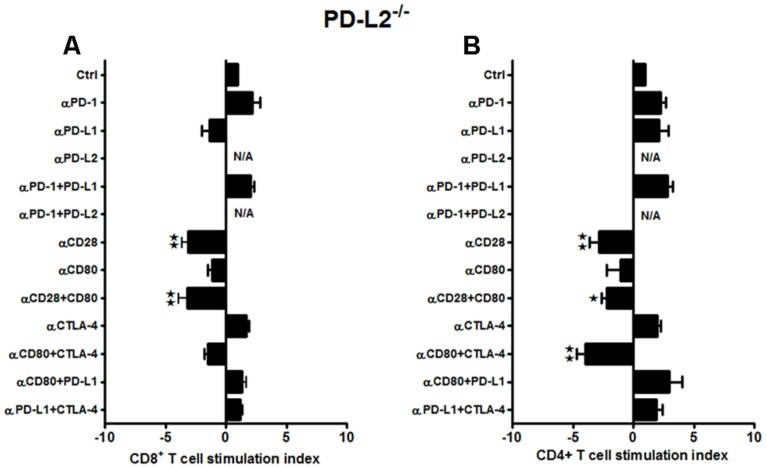
**Subtle changes in the proliferative capacities in the PD-L2^-/-^ mice indicate a minor role of PD-L2 in ischemic stroke.** T-cells were purified, by negative sort, from the spleens of MCAO-subjected PD-L2^-/-^ mice, by labeling with specific microbeads and separating on the AutoMACS^TM^. The purified T-cells were CSFE-labeled and then cultured with non-T-cells from the same mice at a 1:1 ratio (T:APC) in the presence of anti-CD3 antibody (2.5 μg/mL; baseline) and other neutralizing Abs (10 μg/mL) to co-stimulatory molecules, in 96-well plates. After 72 h of culture, cells were washed and evaluated by FACS Calibur for **(A**) CD8^+^ and **(B)** CD4^+^ T-cell expression and CFSE dilution. Data represent the stimulation indices of the CD8 and CD4 T-cells in the presence of neutralizing Abs as compared to the control (T:APC+anti-CD3 Ab) condition). The data are represented such that the baseline value is 1 and all other values are adjusted relative to the baseline. Data presented are representative of splenocytes obtained from seven PD-L2^-/-^ mice, with at least three separate experiments and each experiment comprising duplicates or triplicates of the given neutralizing Ab condition. Significant differences between sample means are indicated as *p ≤ 0.05, **p ≤ 0.01 as compared to the baseline condition.

When similar studies were conducted using splenocytes from MCAO-subjected PD-L2^-/-^ mice, the responses, although more subtle, strongly support results obtained from WT and PD-L1^-/-^ mice. In the absence of PD-L2, proliferation of CD8^+^ T-cells was diminished by treatment with neutralizing anti-CD28 Ab and with the combination of anti-CD28 + anti-CD80 Abs (*p* ≤ 0.01 for each, **Figure 4A**), again implicating CD28/CD80 as the major co-stimulatory pathway that is enabled by co-expression of PD-L1. Similarly, CD4^+^ T-cells demonstrated significantly decreased proliferative responses with anti-CD28 Ab alone (*p* ≤ 0.01) and with the combination of anti-CD28 + anti-CD80 Abs (*p* ≤ 0.05). Moreover, the proliferation response of CD4^+^ T-cells was inhibited by treatment with the combination of anti-CTLA-4 + anti-CD80 Abs (*p* ≤ 0.01, **Figure 4B**), thus implicating a new co-stimulatory pathway that emerged in the absence of PD-L2. Notably, in the absence of PD-L2, there was no significant neutralization of any tested co-inhibitory molecules, particularly PD-1, thus confirming the requirement for PD-L2 in co-inhibitory regulation of proliferation responses for both CD4^+^ and CD8^+^ T-cells.

In summary, these results demonstrate a dominant role for PD-L1 in promoting CD8^+^ and CD4^+^ T-cell proliferation that contributes to increased infarct volumes in mice subjected to MCAO. Our data demonstrate that the presence of PD-L1 promotes the CD28/CD80 co-stimulatory pathway, whereas its absence obviates co-stimulation and allows full expression of the PD1/PD-L2 co-inhibitory pathway as well as the emergence of the otherwise silent CTLA-4/CD80 co-inhibitory pathway, both of which regulate T-cell proliferation in mice subjected to MCAO. In contrast, presence of PD-L2 is required for all co-inhibitory activity, whereas its absence not only reduces expression of the major CD28/CD80 co-stimulatory pathway, but also allows emergence of a second CTLA-4/CD80 co-stimulatory pathway for CD4^+^ T-cells.

### PRESENCE OF PD-L1 ON APCs IS AS IMPORTANT AS IT IS ON T-CELLS, WHILE EXPRESSION OF PD-L2 IS CRUCIAL ON APCs OF WT MICE SUBJECTED TO MCAO

PD-L1 and PD-L2 are the two known ligands for PD-1 (Keir et al., 2008), mostly expressed by APCs. However, they have different expression patterns (Ishida et al., 2002; Yamazaki et al., 2002; Ansari et al., 2003; Iwai et al., 2003; Liang et al., 2003; Salama et al., 2003; Wiendl et al., 2003; Zhong et al., 2007; Keir et al., 2008).

**FIGURE 5:**
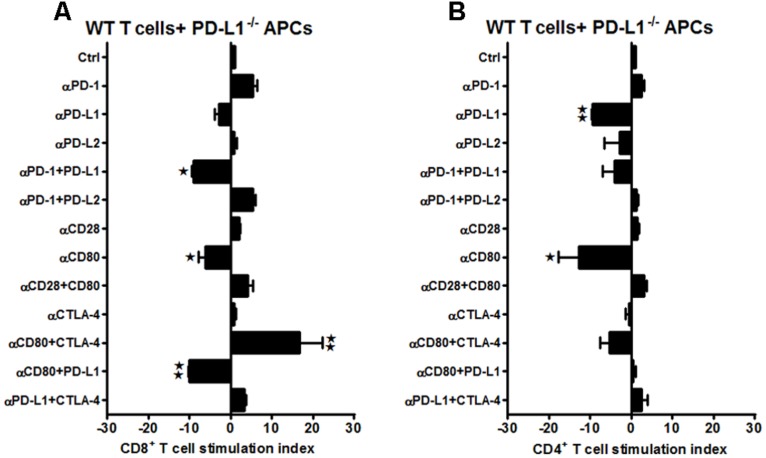
**Presence of PD-L1 on APCs is as important as it is on T-cells, while expression of PD-L2 is crucial on APCs of WT mice subjected to MCAO.** T-cells were purified, by negative sort, from the spleens of MCAO-subjected WT mice, by labeling with specific microbeads and separating on the AutoMACS^TM^. The purified T-cells were CSFE-labeled and then cultured with non-T-cells from PD-L1^-/-^ mice at a 1:1 ratio (T:APC) in the presence of anti-CD3 antibody (2.5 μg/mL; baseline) and other neutralizing Abs (10 μg/mL) to co-stimulatory molecules, in 96-well plates. After 72 h of culture, cells were washed and evaluated by FACS Calibur for **(A)** CD8^+^ and **(B)** CD4^+^ T-cell expression and CFSE dilution. Data represent the stimulation indices of the CD8 and CD4 T-cells in the presence of neutralizing Abs as compared to the control (T:APC + anti-CD3 Ab) condition). The data are represented such that the baseline value is 1 and all other values are adjusted relative to the baseline. Data presented are representative of splenocytes obtained from eight WT, six PD-L1^-/-^, and six PD-L2^-/-^ mice with least three separate experiments and each experiment comprising duplicates or triplicates of the given neutralizing Ab condition. Significant differences between sample means are indicated as *p ≤ 0.05, **p ≤ 0.01 as compared to the baseline condition.

Hence, we further investigated the effects on T-cell proliferation of the expression of PD-L1 and PD-L2 on APC and T-cells obtained from mice post-MCAO. Thus, T-cells were obtained from MCAO-subjected WT mice and labeled with CFSE. To determine if the presence of the PD-ligands is crucial for the antigen presenting cells to influence the proliferative capacities of the T-cells, APCs (non-T-cells) were obtained either from MCAO-subjected PD-L1^-/-^ or PD-L2^-/-^ mice. The WT T-cells and PD-ligand knockout APCs were co-cultured in the presence of various neutralizing Abs to co-stimulatory molecules. As demonstrated in **Figure 5A**, there was a significant decrease in CD8^+^ T-cell proliferation upon using anti-PD-1 (on APC and/or T-cells) + anti-PD-L1 (T-cells only) and anti-CD80 (on APC and/or T-cells) + anti-PD-L1 (on T-cells only) Abs (*p* ≤ 0.05 and *p* ≤ 0.01, respectively) and a similar significant decrease was demonstrated when just anti-CD80 (on APC and/or T-cells) Ab (*p* ≤ 0.05) was used. Based on availability of PD-L1, it follows that the effects of anti-PD-L1 are restricted to the PD-L1^+^ T-cells (not on PD-L1^-/-^ APC), whereas effects of the anti-PD-1 and anti-CD80 Abs could be on the APC or T-cells. It is thus possible that the inhibition of proliferation of CD8^+^ T-cells involves Ab blockade of PD-1 and/or CD80 expressed on APC and PD-L1 expressed only on T-cells. This conceivably could pair CD80/PD-L1 as a possible co-stimulatory pathway for CD8^+^ T-cell proliferation that might be operative in the absence of PD-L1 on APC. Pairing of PD-1/PD-L1 seems unlikely due to many reports to the contrary. Surprisingly, there was a significant increase in proliferation of CD8^+^ T-cells when anti-CD80 + anti-CTLA-4 Abs were used (*p* ≤ 0.01), possibly suggesting a redundant co-inhibitory pathway involving CTLA-4 that might appear in the absence of PD-L1 on APC. However, the increased CD8^+^ proliferation response after treatment of WT cells with anti-PD-1 (**Figure 2A**) was not apparent in the absence of PD-L1 on APC. Similarly, in the CD4^+^ T-cells, besides the significant reduction in proliferation using anti-CD80 (on APC and/or T-cells) Ab (*p* ≤ 0.05), a significant decrease in proliferative capacity was observed when anti-PD-L1 (T-cells only) Ab was also used (*p* ≤ 0.01; **Figure 5B**). However, again, no increase in CD4^+^ proliferation was observed with WT cells in the presence of anti-PD-1 Ab as was observed in **Figure 2B**. Overall, these data demonstrate that the dominant CD28/CD80 co-stimulatory pathway observed in WT and PD-L2^-/-^ mice depends on co-expression of PD-L1, and the current experiments suggest that its expression need be on APCs and T-cells.

**FIGURE 6:**
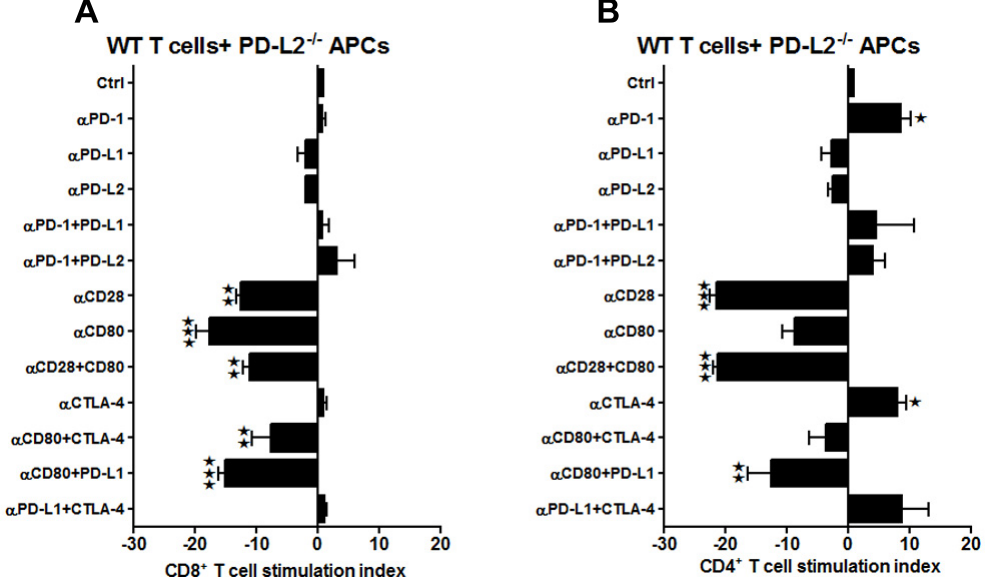
**Presence of PD-L1 on APCs is as important as it is on T-cells, while expression of PD-L2 is crucial on APCs of WT mice, subjected to MCAO.** T-cells were purified, by negative sort, from the spleens of MCAO-subjected WT mice, by labeling with specific microbeads and separating on the AutoMACS^TM^. The purified T-cells were CSFE-labeled and then cultured with non-T-cells from PD-L2^-/-^ mice at a 1:1 ratio (T:APC) in the presence of anti-CD3 antibody (2.5 μg/mL; baseline) and other neutralizing Abs (10 μg/mL) to co-stimulatory molecules, in 96-well plates. After 72 h of culture, cells were washed and evaluated by FACS Calibur for **A** CD8^+^ and **(B)** CD4^+^ T-cell expression and CFSE dilution. Data represent the stimulation indices of the CD8 and CD4 T-cells in the presence of neutralizing Abs as compared to the control (T:APC + anti-CD3 Ab) condition). The data are represented such that the baseline value is 1 and all other values are adjusted relative to the baseline. Data presented are representative of splenocytes obtained from eight WT, six PD-L1^-/-^ and six PD-L2^-/-^ mice with least three separate experiments and each experiment comprising duplicates or triplicates of the given neutralizing Ab condition. Significant differences between sample means are indicated as *p ≤ 0.05, **p ≤ 0.01 and ***p ≤ 0.001 as compared to the baseline condition.

On the other hand, different co-stimulatory molecules seem to play a compensatory role when the APCs lacked PD-L2. The increased proliferation when anti-PD-1 Ab was used with WT T and WT APCs (**Figure 2A**) was lost in CD8^+^ T-cells when the same neutralizing Ab was used in the case of WT T-cells co-cultured with PD-L2^-/-^ APCs. Neutralizing CD28 and CD80 individually or combined, led to a decreased proliferation, both in the CD8^+^ and CD4^+^ T-cells (**Figures 6A,B**, respectively), indicating that in the absence of PD-L2, CD28/CD80 stimulatory interactions are dominant. Collectively, the data indicate that absence of PD-L2 on APCs leads to an over-riding CD28/CD80 interaction that sends positive co-stimulatory signals, leading to increased proliferative capacities of the CD4^+^ T cells obtained from MCAO-subjected WT mice. These data again are in agreement with the need for co-expression of PD-L1 for expression of the CD28/CD80 co-stimulatory pathway.

**FIGURE 7:**
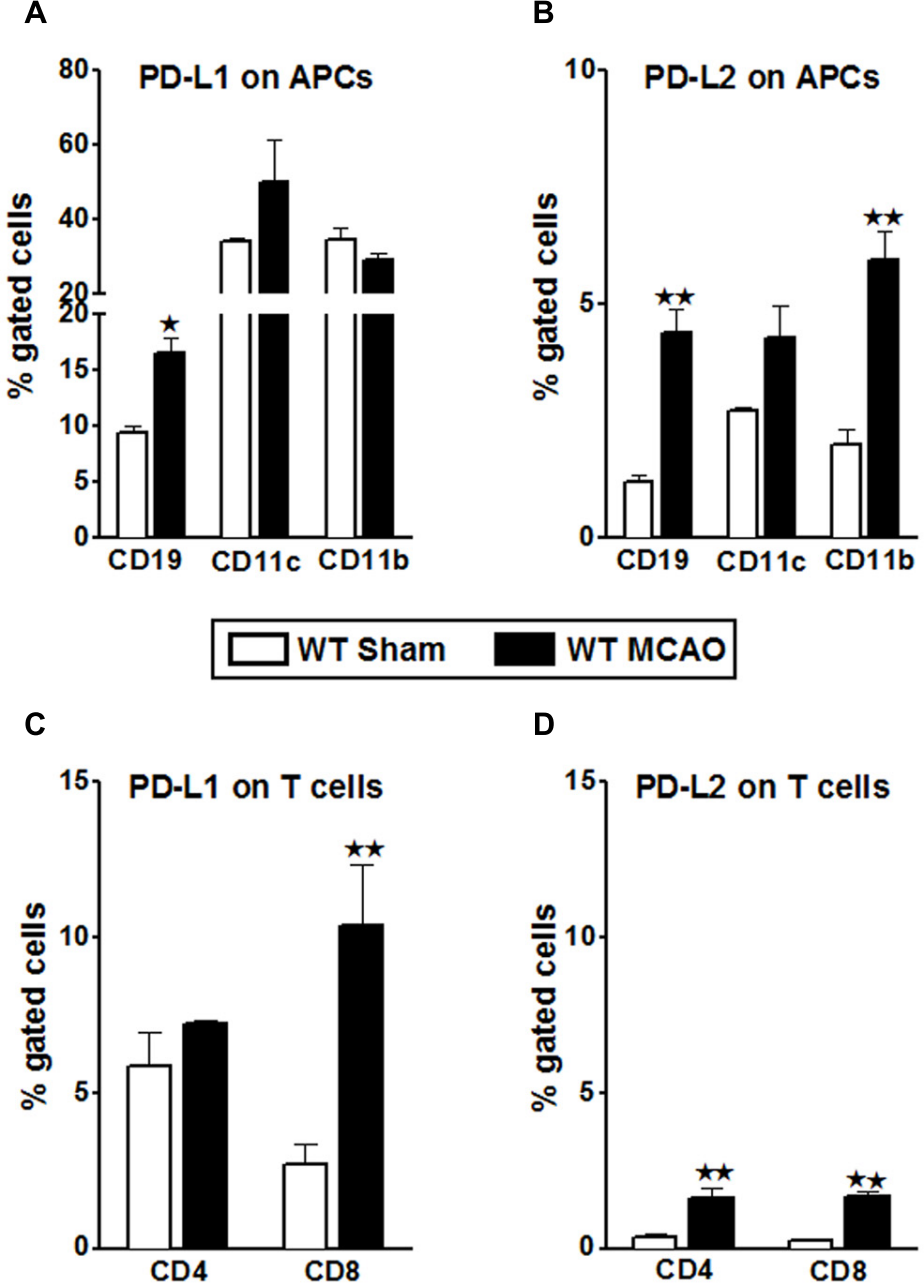
**Characterization of PD-L1 and PD-L2 expression on T-cells and antigen presenting cells of WT splenocytes, post-MCAO.** Splenocytes from sham- and MCAO-subjected WT mice were harvested 96 h after MCAO (60 min) and assessed for expression. **(A)** PD-L1 and **(B)** PD-L2 on APCs; gated on CD19^+^, CD11b^+^, and CD11c^+^ cells. **(C)** PD-L1 and **(D)** PD-L2 on gated CD4^+^ and CD8^+^ T-cells. Values represent mean numbers (±SEM) of indicated cell subsets, gated on live leukocytes (by PI exclusion) from 4–5 mice of each group, from two separate experiments. Statistical analysis was performed using Student’s *t-*test. Significant differences between sample means are indicated as *p ≤ 0.05 and **p ≤ 0.01 as compared to the sham-treated WT mice.

In lieu of the dissimilar proliferative trend exhibited by the WT T-cells when co-cultured with PD-L1^-/-^ APCs, it became important to characterize the changes in PD-L1 and PD-L2 expression on various splenic immune cell types in the WT mice, post-MCAO. In our previous publication (Bodhankar et al., 2013), we demonstrated an increase in the total PD-L2 expression in splenocytes of the PD-L1^-/-^ mice as compared to the WT mice, making a case for a plausible PD-1/PD-L2 co-inhibitory interactions. We also demonstrated that the expression levels of total PD-L1 in both the WT and PD-L2^-/-^ mice remained similar after MCAO. Hence, splenocytes from sham- and MCAO-subjected WT mice were isolated and the percent expression of PD-L1 and PD-L2 on various immune cell types was determined by flow cytometry. It is known that PD-L1 has much broader expression pattern than PD-L2 and it is also characterized to be present on T-cells in addition to APCs. Hence, we also evaluated PD-L1 and PD-L2 expression on T-cells. The PD-L1 expression was significantly increased on the CD8^+^ T-cells (*p* ≤ 0.01) with a trend towards increased expression in the CD4^+^ T-cells, after stroke in WT splenocytes (**Figure 7C**). Even if the T-cells (CD4^+^ and CD8^+^ T-cells) demonstrate an increase in expression of PD-L2 after stroke (**Figure 7D**), the level of expression is only a fraction compared to that of PD-L1 on T-cells. Results upon assessing the expression of PD-L1 on the classic APCs demonstrated that the expression of PD-L1 increased significantly (*p* ≤ 0.05) only on CD19^+^ B-cells with a trend towards increased expression on CD11c^+^ dendritic cells (**Figure 7A**). There was, however, no change in the PD-L1 expression on the CD11b^+^ monocytes after MCAO as compared to levels expressed in spleens of sham-treated WT mice. Similarly, when the expression levels of PD-L2 were assessed on the classic APCs, its expression was significantly increased on the CD19^+^ B cells (*p* ≤ 0.01) and CD11b^+^ monocytes (*p* ≤ 0.01), with a trend in increased expression on the CD11c^+^ DCs (**Figure 7B**). Thus, the abundance of expression of PD-L1 on the CD8^+^ and likely the CD4^+^ T-cells justifies the decrease in the proliferative responses of the WT T-cells upon co-culture with PD-L1^-/-^ APCs in presence of anti-PD-L1 Ab (CD4^+^ T-cells; **Figure 5B**) and anti-PD-1 + anti-PD-L1 and anti-CD80 + anti-PD-L1 conditions (CD8^+^ T-cells; **Figure 5A**). Overall, these results indicate that the presence of PD-L1 on T-cells is as crucial as it is on the APCs, to elicit its functions after stroke.

### BREGS DECREASE INFARCT VOLUMES IN MALE PD-L2^-/-^ MICE, BUT ARE DISPENSABLE WHEN TRANSFERRED TO PD-L1^-/-^ MICE 4 H AFTER MCAO

As demonstrated in **Figures 6A,B** and **7B**, the presence of PD-L2 on classic APCs is crucial. Moreover, as elucidated in **Figure 7B**, PD-L2 expression was significantly increased on CD19^+^ B cells. Also, our previous study (Bodhankar et al., 2013) demonstrated that even though the PD-L1^-/-^ and PD-L2^-/-^ mice had smaller total infarct volumes compared to the WT mice, the pro-inflammatory status as far as infiltrating cells in the brains was the lowest in the PD-L1^-/-^ as compared to both the WT and PD-L2^-/-^ mice. Also, the PD-L2^-/-^ mice had an intermediary pro-inflammatory status, with immune parameters trending to be more like the WT mice rather than that like PD-L1^-/-^ mice. Recently, we demonstrated (Bodhankar et al., 2014) that transfer of IL-10^+^ B cells markedly reduced infarct volumes in WT recipient mice when given 4 h after MCAO. Hence, in an attempt to discern the regulatory mechanism pertaining to these PD-ligands, we hypothesized that similar to WT recipients of IL-10^+^ B cells (regulatory B cells; Bregs), 4 h after MCAO, the PD-L2^-/-^ recipients of IL-10^+^ B cells, would be protected, thus indicating that the presence of PD-L2 on B cells is indispensable for the B cells’ ability to mediate its immune-modulatory actions. Thus, we examined the relative contribution of PD-L1 and PD-L2, when present on the Bregs, in mediating their protective properties.

**FIGURE 8:**
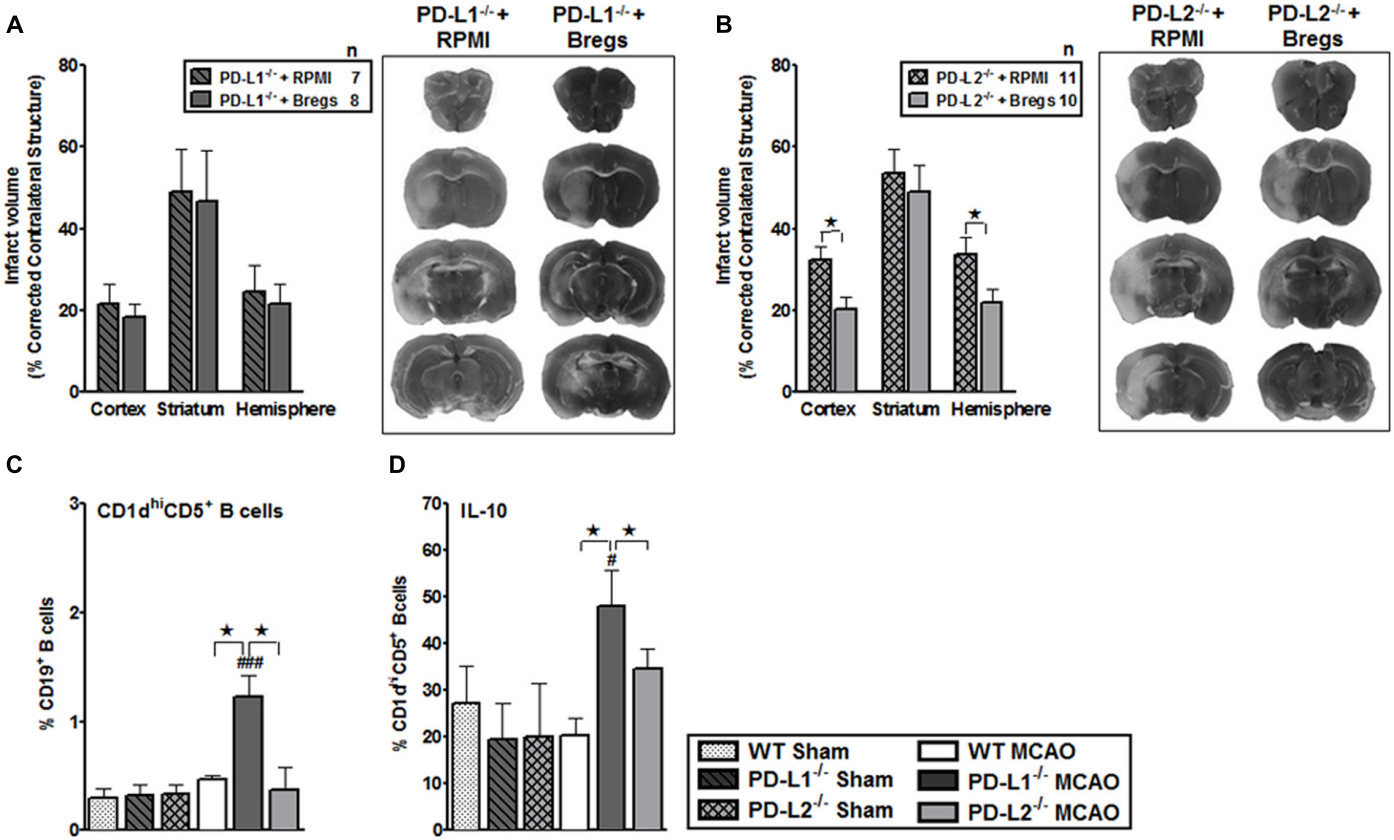
**Bregs decrease infarct volumes in male PD-L2^-/-^ mice, but are dispensable when transferred to PD-L1^-/-^ mice 4 h after MCAO. (A)** Intravenous transfer of 5 million IL10^+^ B-cells 4 h after surgery to induce MCAO in PD-L1^-/-^ mice 96 h following 60 min of MCAO compared to intravenous transfer of RPMI vehicle (no cells) and its representative 2,3,5-triphenyltetrazolium chloride (TTC) stained cerebral sections 96 h following 60 min of MCAO. **(B)** Intravenous transfer of 5 million IL10^+^ B-cells 4 h after surgery to induce MCAO in PD-L2^-/-^ mice 96 h following 60 min of MCAO compared to intravenous transfer of RPMI vehicle (no cells) and its representative TTC stained cerebral sections 96 h following 60 min of MCAO. Significance values represent mean ± SEM. *p < 0.05, **p < 0.01. Splenocytes from sham-treated and MCAO-subjected WT, PD-L1^-/-^ and PD-L2^-/-^ mice were harvested 96 h after MCAO (60 min) and assessed for expression of: **(C)** CD1d^hi^CD5^+^CD19^+^ (*Breg*) cells and **(D)** IL-10 production by gated Breg cells. Data are representative of two independent experiments with spleens processed from four to five individual mice (mean ± SEM). Significant differences between sample means are indicated (*p ≤ 0.05 compared to the PD-L1^-/-^ mice, post MCAO). Significant differences within the strains are indicated (^#^*p* ≤ 0.05 and ^###^*p* ≤ 0.001 as compared to its respective sham-treated counterparts).

B cells obtained from spleens of IL-10-GFP reporter mice were purified by negative selection and cultured *in vitro* for 48 h in presence of LPS. This *in vitro* culture enriched the B cells to produce high amounts of IL-10 cytokine. These IL-10-enriched Bregs were transferred to PD-L1^-/-^ and PD-L2^-/-^ recipients. As shown in **Figure 8A**, PD-L1^-/-^ mice that received IL10^+^ B-cells (*n* = 8) 4 h after MCAO exhibited no differences in the infarct volumes in each of the cortex, striatum and hemisphere regions after 60 min MCAO followed by 96 h of reperfusion compared to no-cell transferred vehicle (RPMI) controls (*n* = 7). However, when IL-10^+^ B-cells were transferred to the PD-L2^-/-^ mice (*n* = 10) 4 h after MCAO, there was as significant reduction in cortical (*p* ≤ 0.05) and total hemisphere (*p* ≤ 0.01) infarct volumes after 60 min MCAO followed by 96 h of reperfusion compared to no-cell transferred vehicle (RPMI) controls (*n* = 11). Representative cerebral sections from PD-L1^-/-^ and PD-L2^-/-^ mice treated with RPMI or IL10^+^ B-cells are shown in **Figures 8A,B**.

We further ascertained whether differences in surface expression of CD1d^hi^CD5^+^ on B cells (Bregs) in the PD-L1^-/-^ or PD-L2^-/-^ mice as compared to WT mice might reflect the differences in protection in these strains, after the transfer of Breg cells. We assessed the expression of a recently characterized regulatory B-cell sub-population, known as the CD1d^hi^CD5^+^CD19^+^ Bregs (Bodhankar et al., 2011) which is known to effectively down-regulate T-cell activation by virtue of their IL-10 production (Yanaba et al., 2008). We determined the CD1d^hi^CD5^+^ expression (**Figure 8C**) and IL-10-secretion by B cells (**Figure 8D**). Splenocytes from sham- and MCAO-subjected WT, PD-L1^-/-^ and PD-L2^-/-^ mice were obtained and the expression of cell surface molecules, CD1d and CD5, was determined on CD19^+^ B cells (**Figure 8C**) as was the intracellular IL-10 expression (**Figure 8D**). As demonstrated in **Figures 8C,D** a significant increase in the percentage of not only CD1d^hi^CD5^+^CD19^+^ Bregs but also the production of IL-10 by this cell sub-type was observed to be already present in the PD-L1^-/-^ mice as compared to both WT (*p* ≤ 0.05), and the PD-L2^-/-^ (*p* ≤ 0.05) mice. Thus, these results demonstrate that Bregs decrease infarct volume in male PD-L2^-/-^ mice, but are dispensable and cannot further affect the ongoing infarction process when transferred 4 h after MCAO to PD-L1^-/-^ knockout mice that already have increased levels of Breg cells.

## DISCUSSION

Stroke remains the third leading cause of death in adults worldwide and the most frequent cause of permanent disability in the world (Donnan et al., 2008). Although underlying mechanisms have not been completely unraveled, that ischemia evokes inflammatory responses has been well characterized. Besides the well-defined players of innate immunity, which infiltrate the damaged brain area after ischemic stroke, our past studies demonstrated that the T-cells from blood and lymph nodes secrete increased levels of inflammatory cytokines after activation following ischemic stroke (Offner et al., 2006). Also a significant increase in T lymphocytes in the ischemic hemisphere has been demonstrated at 3 days post-reperfusion (Gelderblom et al., 2009). Our group has also demonstrated using SCID mice that T lymphocytes have a damaging effect on early ischemic brain injury (Hurn et al., 2007). However, the mechanisms of T-cell-mediated brain injury following stroke are currently unclear. It is not clear whether the activation of T-cells follows the classical antigen-dependent pathway or if the infiltration into the brain following stroke is too rapid to follow the classical pathway. A recent study by Kleinschnitz et al. (2010) demonstrated that the antigen-dependent activation of T-cells is not required for them to contribute substantially to the infarct volume present at 22 h after ischemic stroke. However, several studies also demonstrate that previously activated T lymphocytes, i.e., due to preexisting infection, cardiovascular disease (Guzik et al., 2007; Andersson et al., 2010) or even in autoimmune disease (Ren et al., 2012) cause additional damage in the brain, following ischemic stroke. Furthermore, two more studies indicate that administration of the recombinant TCR ligand, RTL551 linked to a CNS antigen (which blocks classical antigen-dependent T-cell activation), resulted in a reduced infarct volume following ischemic stroke (Subramanian et al., 2009; Dziennis et al., 2011). These findings suggest that an adaptive immune response to brain antigens occurred following stroke, and that classical T-cell activation may indeed have contributed to post-ischemic brain damage. Moreover, tolerance against brain antigens by mucosal administration of a CNS myelin antigen before stroke has been reported to improve outcome after stroke (Becker et al., 1997; Frenkel et al., 2005; Gee et al., 2008), further suggesting that antigen-dependent lymphocyte activation occurs following stroke, and that it contributes to brain injury. However, the mechanism(s) of antigen-independent T-cell “activation” within hours after stroke are currently unknown.

T-cell activation involves the B7 family of co-stimulatory molecules, which provide pivotal stimulatory or inhibitory signals and a balance between these signals is required for effective immune responses to various stimuli. Our past study (Ren et al., 2011) on one hand demonstrated that PD-1 is crucial in mediating protection in ischemic stroke, but on the other hand, our subsequent study (Bodhankar et al., 2013) involving PD-ligand knockout mice demonstrated the stimulatory role of the PD-ligands in ischemic stroke. Hence, it was necessary to investigate the nature of interactions between PD-1 and both its ligands for understanding the susceptibility, pathogenic mechanisms, and protection afforded after ischemic stroke. In another previous study (Bodhankar et al., 2013), we demonstrated that WT and PD-L2^-/-^ mice demonstrated a significantly increased expression of the co-stimulatory molecule, CD80 (B7.1) on the CD11c^+^ dendritic cells and by the CD11b^+^ monocytes as compared to that in the PD-L1^-/-^ mice, after MCAO. Also, the expression levels of total PD-L1 on splenocytes of both the WT and PD-L2^-/-^ remain similar after MCAO. Thus, we speculated that the CD80-CD28 interaction overrides the CD80-CTLA-4 or CD80-PD-L1 interactions leading to T-cell activation in the WT and PD-L2^-/-^ mice. Conversely, low CD80 expression by the APCs in PD-L1^-/-^ mice suggested T-cell signaling through CTLA-4, leading to a suppressor phenotype. Also an increase in the total PD-L2 expression in the PD-L1^-/-^ mice as compared to the WT mice, made a case for a plausible PD-1/PD-L2 co-inhibitory interaction in the absence of PD-L1. Hence the purpose of this study was to verify the aforementioned hypotheses. In order to do so, we first determined the change in the expression levels of some of the T-cell-related major players of the co-stimulatory pathway in WT mice, post-stroke. CD28 has a predominant role during initial T-cell activation while ICOS regulates antigen-experienced T-cells, but CD28 and ICOS synergize to promote the activation of T-cell responses (Greenwald et al., 2005). Also, CTLA-4, an immune inhibitory receptor within the CD28 family of co-stimulatory molecules (Salomon and Bluestone, 2001; Greenwald et al., 2005; Peggs et al., 2009), shares its ligands B7-1 and B7-2 with CD28 but binds them with differential kinetics (Riley and June, 2005). CTLA-4 is induced in activated T-cells and inhibits T-cell activation by engaging specific signaling pathways and by out-competing the positive co-stimulatory receptor CD28 (Salomon and Bluestone, 2001; Engelhardt et al., 2006). It is also constitutively expressed in FoxP3^+^ Tregs (Read et al., 2000; Annunziato et al., 2002). Hence, the levels of expression of these three players were determined on splenocytes from sham- and MCAO-subjected WT mice. But as demonstrated in **Figures 1A–C**, the expression levels of CD28 are significantly increased and those of CTLA-4 were reduced; however, the expression of ICOS did not change between sham and MCAO-subjected mice until day 3 post-stroke. Thus, it appeared as though the molecules of the CD80/CD28, CTLA-4, and the PD-1/PD-L pathway are more critical than others in ischemic stroke. Therefore, we next focused on these co-stimulatory players and how the preferential neutralization of each these players influenced the proliferative capacities of the T-cells of the WT mice, post-stroke.

Our speculation of the over-riding association between CD80 and CD28 seems to hold true, more so, in case of CD8^+^ T-cells of WT mice as demonstrated in **Figure 2A** because the proliferative capacities of CD8^+^ T-cells were significantly decreased in presence of anti-CD80 and anti-CD80 + anti-CD28 neutralizing Abs, whereas neutralizing PD-1 significantly increased proliferation. In support of our findings, studies involving intrahepatic virus-specific T-cell dysfunction, particularly in HCV-infected liver also demonstrate that T-cell function can be synergistically reversed by combined PD-1/CTLA-4 blockade *in vitro* in a CD4-independent and CD28-dependent manner (Nakamoto et al., 2009). The study demonstrated that functional response to PD-1/CTLA-4 blockade was abolished in HCV-specific CD8^+^ T-cells by CD28-depletion suggesting that immune exhaustion at the site of antigen expression may be reversed by combined inhibitory receptor blockade. In another study, it has also been suggested that since PD-L1 also interacts with B7-1 (Butte et al., 2008), both anti-PD-L1 and anti-CTLA-4 can increase the accessibility of B7-1 to CD28 (Parry et al., 2005). Thus, our findings in this current study are in synchrony with the aforementioned studies, since as demonstrate in **Figures 2A,B** CD8^+^ T-cells seem to be impacted with CD28/CD80 interactions while CD4^+^ T-cells with PD-1/PD-L2 interactions.

**FIGURE 9:**
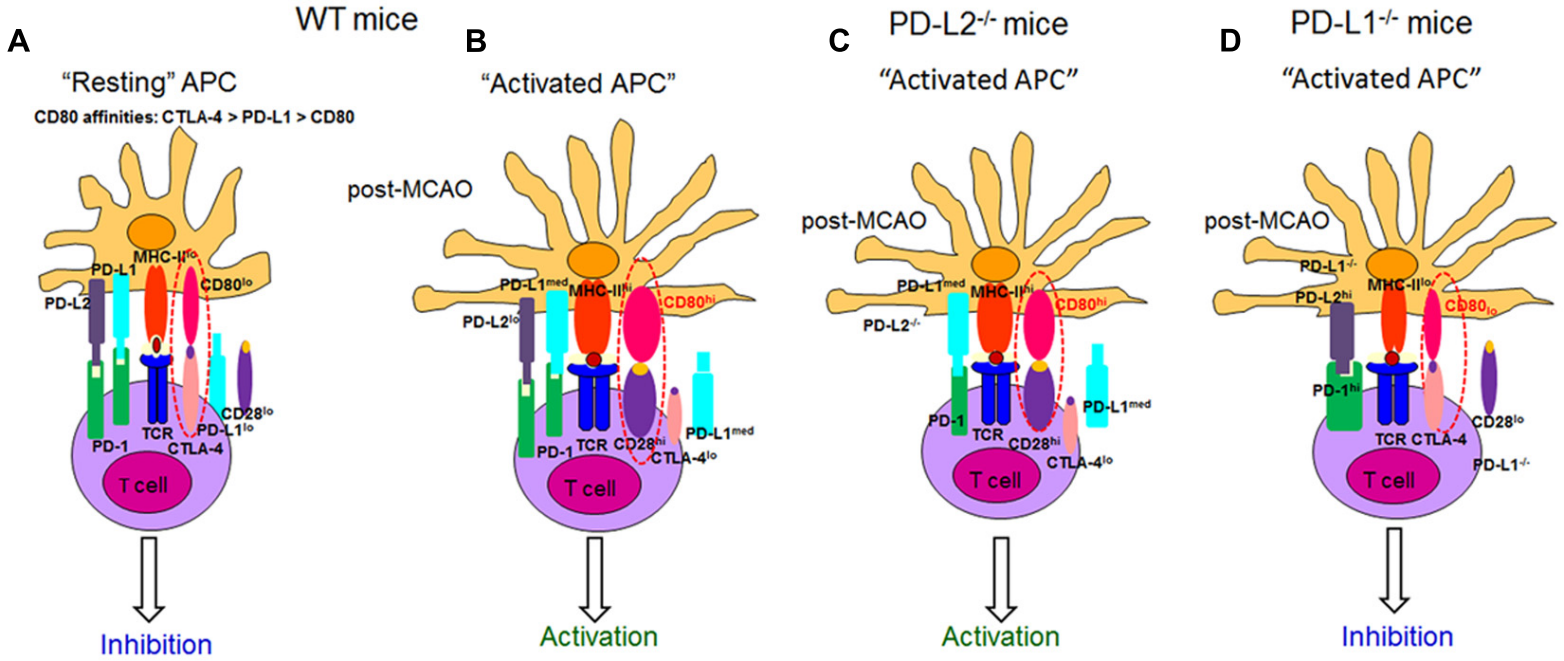
**Proposed model for the role of CD28, CTLA-4, CD80, and the PD-1/PD-L pathway in mice after ischemic stroke.** The following mechanisms of action for the various co-stimulatory molecules belonging to the B7 family are postulated. The affinity of CD80 for CTLA-4, PD-L1, and CD28 are *k*_d_ = 0.4, 1.4, and 4.0 μM, respectively. Thus, at homeostatic levels, **(A)** PD-L1 competes with CTLA-4 for CD80 binding, thus providing in concert with MHC molecules a non-activating signal that promotes naïve T-cell survival, while simultaneously suppressing T-cell responses by outcompeting signaling through CD80/CD28. When stroke is induced in **(B)** WT and **(C)** PD-L2^-/-^ mice, the level of CD80 expression is significantly increased, enhancing T-cell signaling through CD28 and further promoting T-cell activation resulting in an effector phenotype. However, when stroke is induced in the absence of PD-L1. **(D)** PD-L1^-/-^ mice, with low CD80 expression and without PD-L1 to compete with CTLA-4, T-cell signaling results through CTLA-4. Also, MCAO-subjected PD-L1^-/-^ mice exhibit significantly higher levels of both PD-1 and PD-L2, resulting in their prominent interactions. Thus, we propose that the CD80/CTLA-4 and PD-1/PD-L2 eventually lead to a suppressor phenotype. *hi* high expression, *lo* low expression, *med* medium expression, *CTLA-4* cytotoxic T lymphocyte-associated antigen-4, *TCR* T-cell receptor, *MHC* Major Histocompatibility complex (adapted from Zozulya et al., 2010).

PD-L1 and PD-L2 are known to mediate both positive and negative signals. Contrary to the best-characterized inhibitory role for PD-L1, a stimulatory role has been suggested in a number of recent studies. For example, transgenic over-expression of PD-L1 on pancreatic beta cells enhanced autoimmunity instead of suppressing it (Subudhi et al., 2004). As a result of PD-L1 over-expression in beta cells, CD8^+^ T-cell proliferation was enhanced and immunological tolerance was broken, as mice developed spontaneous diabetes (Subudhi et al., 2004). In yet another study (Waisman and Yogev, 2009), an unexpected beneficial effect from PD-L1^-/-^ DC was demonstrated where intra-cerebral microinjections resulted in amelioration of subsequent EAE (Waisman and Yogev, 2009). Furthermore, our results in **Figures 3A,B** also demonstrate that the most impact on the proliferative capacities of both the CD8^+^ and CD4^+^ T cells in PD-L1^-/-^ mice, after stroke, was when neutralizing Abs to CD80 + CTLA-4 and PD-1 + PD-L2 were used. These data indicate that in the absence of PD-L1, with the decreased expression of CD80, CTLA-4/CD80 interactions become prevalent, leading to the induction of inhibitory signals in T cells. We thus propose a working model based on all our results obtained thus far (**Figure 9**).

Several lines of work have also suggested that the broad expression of PD-L1 in lymphoid and non-lymphoid organs and the more restricted, but overlapping, expression of PD-L2 in DC and macrophages may explain, in part, how these B7 family members can have overlapping and/or distinct biological functions (Loke and Allison, 2003). Several animal models suggest that distinct functions could be elicited by PD-L1 and PD-L2 (Liang et al., 2006; Zhu et al., 2006). Recent data suggest higher affinity binding of PD-L2 to PD-1 (Lazar-Molnar et al., 2008). Hence, the ability of PD-L1 and PD-L2 to compete for PD-1 might be important to consider especially on cells that are known to express both ligands, such as APCs, but also in tissues undergoing inflammation (Greenwald et al., 2005). However, differences remain relative to binding kinetics and expression levels. In general, PD-L2 is expressed late, and at lower levels. Data suggest that the cytokine environment may have an important role in differentially regulating PD-L1 and PD-L2 expression and modulating inflammatory responses in the lung microenvironment. In fact, studies also show that PD-L1 and PD-L2 have important but opposing roles in modulating and polarizing iNKT-cell function in airway hyper-responsiveness (AHR) and airway inflammation (Akbari et al., 2010). Shin et al. (2005) have shown that PD-L2, but not PD-L1 was found to elicit direct activating effects on DCs and this effect is supposed to enhance immune responses. However, the concurrent presence of PD-L1 on the same cell prevented this activating effect of PD-L2 due to competition with PD-1 availability (Ghiotto et al., 2010). Hence, these studies support our findings that in the absence of PD-L1, the PD-1/PD-L2 pathway is dominant and pivotal in affecting the proliferative responses (**Figures 3A,B**). At the same time, only subtle changes in proliferative capacities were exhibited by both CD8^+^ and CD4^+^ T-cells obtained from PD-L2^-/-^ mice (**Figures 4A,B**), wherein PD-L1 expression is intact. These data imply that a dominant pathogenic role is played by PD-L1 in ischemic stroke as compared to the other PD-1 ligand, namely PD-L2. Interestingly, our study further demonstrated the stimulatory role of PD-L1, especially when WT T-cells were co-cultured with PD-L2^-/-^ APCs, in that the presence of PD-L1 on T cells is critical as indicated by the fact that when CD80 (APC-expressed) and PD-L1 (expressed on T-cells in this scenario) neutralizing Abs were used, a decrease in the proliferative capacities of the CD8 (**Figure 6A**) and CD4 (**Figure 6B**) T-cells was demonstrated, thus implicating these factors as co-stimulatory molecules. Furthermore, when we extended our studies to decipher the cell type responsible to mediate the critical function of immune-suppression or immuno-activation by each of PD-ligands, post-stroke, we demonstrated a significant increase in PD-L2 on splenic APCs of WT mice (**Figure 7B**) especially CD19^+^ B cells. Also, lower infarct volumes in the PD-L2^-/-^ recipients upon reconstitution with PD-L2^+^ IL-10-enriched Bregs were demonstrated. Thus, the PD-L2^-/-^ recipients are protected like the WT mice that receive IL-10^+^ Bregs, 4 h after MCAO (Bodhankar et al., 2014). However, no difference in the infarct volumes was demonstrated in the PD-L1^-/-^ recipients of PD-L1^+^Bregs. Collectively, these findings indicate the crucial role of the PD-L2 on the APCs, especially Breg cells (**Figures 6, 7B** and **8A**), in contrast to a dispensable role of PD-L1 on the Bregs. In the context of possible therapy, the data support the prediction that treatment with anti-PD-L1 Ab would likely be beneficial for nullifying CD8^+^ and CD4^+^ T-cell effects by reducing the co-stimulatory CD28/CD80 interactions and enabling co-inhibitory PD-1/PD-L2 and CTLA-4/CD80 interactions. On the other hand, neutralization of PD-L1 would appear to reduce or obviate regulatory effects of Breg cells on stroke. Subsequent experimentation will thus be required to sort out the safety and relative efficacy of antibody blockade of PD-L1 vs. transfer of Breg cells in the presence of functional PD-L1.

In summary, the current study conclusively demonstrates for the first time that PD-L1 and PD-L2 have distinct roles in controlling the T-cell activation after ischemic stroke. CD80/CD28 interactions played a prominent regulatory role for the CD8^+^ T-cells and the PD-1/PD-L2 interactions were dominant in controlling the CD4^+^ T-cell responses in WT mice, after stroke. A suppressive phenotype in PD-L1 deficient mice was attributed to CD80/CTLA-4 and PD1/PD-L2 interactions. PD-L2 was crucial in modulating CD4^+^ T-cell responses, whereas PD-L1 regulated both CD8^+^ and CD4^+^ T-cells. To establish the contribution of PD-L1 and PD-L2 on regulatory B-cells (Bregs), infarct volumes were evaluated in male PD-L1- and PD-L2-deficient mice receiving IL-10^+^ B-cells 4 h after MCAO. PD-L2- but not PD-L1-deficient recipients of IL-10^+^ B-cells had markedly reduced infarct volumes, indicating a regulatory role of PD-L2 on Bregs. Overall, it is apparent that these pathways provide redundant positive and negative signals and that there is some hierarchy in the orchestration of their signals. The current study provides insights into mechanisms of T-cell activation in ischemic stroke, thus exhibiting the potential for therapeutic intervention for controlling T-cell responses. Our results clearly imply that PD-L1 and PD-L2 differentially control induction of T- and Breg-cell responses after MCAO, thus suggesting that selective targeting PD-L1 and PD-L2 might represent a valuable therapeutic strategy in stroke.

## AUTHOR CONTRIBUTIONS

Sheetal Bodhankar designed and performed the immunology experiments, carried out statistical analyses, prepared graphics and wrote the manuscript; Yingxin Chen performed the MCAO procedures, carried out statistical analyses and prepared the graphics for the infarct volume representation; Arthur A. Vandenbark critiqued and edited the manuscript; Stephanie J. Murphy directed study design and data analysis of the MCAO experiments and edited the manuscript; Halina Offner directed the overall study, designed and supervised the immunological studies and data analysis and edited the manuscript. All authors read and approved the final version of the manuscript.

## Conflict of Interest Statement

The authors declare that the research was conducted in the absence of any commercial or financial relationships that could be construed as a potential conflict of interest.
